# Morbidity profile of orphan children in Southern India

**DOI:** 10.18203/2349-3291.ijcp20183537

**Published:** 2018

**Authors:** Shraddha Toutem, Vanya Singh, Enakshi Ganguly

**Affiliations:** Department of Community Medicine, Mediciti Institute of Medical Sciences, Medchal Mandal, Telangana, India

**Keywords:** Children, India, Morbidity, Orphan, Treatment seeking

## Abstract

**Background:**

Orphan children globally and in India are increasing. Magnitude of their health problems is unknown. The present study was carried out to assess the morbidity pattern of orphan children aged 10–16 years, assess their knowledge about signs and symptoms of common childhood morbidities and treatment-seeking practices.

**Methods:**

One hundred institutionalized orphan children aged 10–16 years were studied for 6 months. Data was collected by trained investigators regarding sociodemographic background, awareness about common morbidities and treatment seeking practices. Thorough clinical examination and anthropometric measurements were done. Distribution of morbidities was shown.

**Results:**

80% of the boys and 68% of the girls had low BMI. 78% suffered from multiple morbidities of which 76% had infections of skin and appendages while 74% had ear problems. 26% had diarrhea and 21% had B-complex deficiency. The mean duration of all diseases was 7±1.1 days. Awareness about diseases and their complications was low; and self-care was highly prevalent for most health problems.

**Conclusions:**

Orphan children in South India suffer from many morbidities about which their awareness and treatment seeking is low.

## INTRODUCTION

The number of orphans is increasing globally with their population estimated to be 140 million in 2015 worldwide.^1^ These children, who have lost either or both parents, are frequently sent to orphanages where they are deprived of parental care and, often, food, education, and medical care.^2^ UNICEF reported 25,000,000 orphan children (from all causes) present in India, the highest in South East Asian region in 2007.^3^

Research focusing on various age groups of such children has mostly reported high prevalence of multiple infectious conditions and co-morbidities.^4^ Poor nutritional status has been shown to be widely prevalent and directly associated to morbidities among institutionalized children. The number of destitute children in our country seeking admission to orphanages is on the rise due to various social, political and economic factors.^5^ Hyderabad, a South Indian city has more than 100orphanages (about 24 of whom are registered), each housing 23–25 orphan children on average aged 3 to 18 years.

Studies often report that lack of parental care and denial of a secure family life, coupled with congested and often unhygienic living conditions, affect orphan children adversely.^6,7^ Though in recent years orphanages have started receiving attention through government as well as Non- Government Organizations, there is conclusive evidence from the Western world about the detrimental effects of institutionalized care on children, which is directly proportional to the length of stay.^8,9^ Research identifies the risks of orphanage care that may broadly be classified as:

- Infectious morbidity,
- Nutrition and growth,
- Cognitive development,
- Socio-affective development,
- Physical and sexual abuse in the institution, all of which are more during infancy and early years of life.^10^

The uncertainty abounding the efficiency of these organizations, especially in third world countries, have led researchers to devise proxy indicators for estimating efficiency and quality through assessment of health status, among other indicators, of the residents.^11^ A valid measure, required for planning promotional measures for the welfare of orphaned children, requires reliable data on their health and nutrition status. Such data was grossly deficient from different parts of the country, more so from South India. It, therefore, was imperative to collect data of good quality so that the same might be used by policy-makers for designing programs to improve need-based service delivery by the orphanages. The present study was designed to study the impact of institutional care offered by orphanages by assessing the health and nutritional status of the resident orphaned children in the city of Hyderabad in India. The objectives of this paper were to assess the morbidity status of children aged 10–16 years residing in orphanages for more than six months, and to assess the knowledge of the orphan children about signs and symptoms of common childhood morbidities and their treatment-seeking practices.

## METHODS

A cross-sectional study was done from 1^st^ August 2014 through 31^st^ January 2015. Institutional Ethical committee provided clearance for conducting the study. All orphanages in Hyderabad city were listed and contacted through telephone to ascertain the number and age group of the orphaned children residing in the facility. At least 20% orphanages were randomly selected for inclusion in the study. The head of the institution (orphanage) was contacted and the purpose of the study was explained to obtain an informed written consent. At least 20% of the children meeting the inclusion criteria were randomly selected from each selected institution and asked to provide assent for participating in the study. The inclusion criteria were consenting children aged 10–16 years living and studying in an orphanage in Hyderabad for more than six months. Children currently admitted in hospital for serious illness or those who had not stayed in the orphanage for six months were excluded.

One hundred orphaned children from five orphanages in Hyderabad city were thus, randomly selected using a computer-generated random number table, as per feasibility. Data was collected by two trained investigators, who received training for two days in the Department of Community Medicine on the study protocol and delivering the questionnaires and performing physical examination according to standard protocol. The questionnaire was developed to yield demographic and morbidity-related information about the participants. Details related to number of days child was suffering from present condition, number of episodes of illnesses in past one year, number of times adequate treatment was offered, and place of treatment were collected. Anthropometric examination was conducted for each participant and recorded.

### Body weight

Weight was recorded to the nearest multiple of 10 grams using an electronic weighing balance, Tanita HD −318 digital weighing scales (Tanita Corporation, Tokyo, Japan). Each participant was weighed with minimum clothing and without shoes, standing upright with arms hanging by the sides.

### Height

Height was recorded to the nearest centimeter (cm) using a non-flexible fibreglass tape, Seca 206 mechanical measuring tape (Seca, GmBH and Co Kg, Hamburg, Germany). The respondent was asked to stand upright on a firm level ground, against a flat vertical surface without shoes.

### BMI calculation

The body mass indices of the participants were calculated in kg/m^2^ based on the measured weight and height and were plotted on separate BMI charts for boys and girls.

### Clinical assessment

The condition of skin, eyes, hair, gums, nails and thyroid gland was observed for clinical assessment of nutritional deficiencies. Systemic examination for reported morbidities was conducted according to the hospital protocols of Mediciti Hospital.

### Statistical analysis

Data was analyzed using SPSS 21 software. All quantitative data was reported in percentages. Means and standard deviations for continuous data were reported. T test was done to report differences between males and females. P< 0.05 was considered significant.

## RESULTS

One hundred children were studied; the response rate was 100%. The demographic characteristics are shown in Table 1. The common morbidities reported are shown in Table 2. Skin infections were reported most commonly followed by common cold and fever. Infections of the eye were the lowest in prevalence. Knowledge of sign and symptoms of common childhood morbidities was low among resident children, especially for fevers, skin infections/ infestations and ear conditions. None of the children knew the common conditions presenting with fever (including malaria, dengue, typhoid and others).

None of the children had heard about scabies earlier, and none considered otitis media to be a dangerous condition. Only two children who had previous pneumonia knew about common symptoms of the disease, but not complications. None of the children were aware that worm infestation could occur through faeco-oral route. Treatment seeking practices for the orphans for selected morbidities are shown in Table 3. For other diseases/conditions, medical help was not sought.

## DISCUSSION

Ours is amongst the very few Indian studies done upon orphanage children to report the morbidities and treatment seeking amongst them. The high prevalence of common morbidities reported in the present study as well as the inadequate treatment seeking practices of orphanage heads reflects the poor living conditions and inadequate knowledge of common diseases and their treatment. It also shows the poor utilization of healthcare facilities, although all had access to a doctor; the reasons for which remain unexplored. We also found the mean number of days of suffering with illness and mean number of illness episodes in a year to be higher among younger age groups (not shown in table), which is easily explained by lower immunity at younger ages compared with older children.

A previous study done upon orphans in the Southern state of Tamil Nadu similarly reported high prevalence of skin infections including pediculosis (25%) and oro-dental problems including dental caries (50%).^12^ Similar prevalence of ear conditions was also reported. They however, also reported ophthalmic problems including Bitots spots which we did not find in our population. While these differences may be explained by the semantic and climatic variations between the two regions, the fact that orphan children are more prone to multiple co-morbidities, compared with general population of children, remains true. Vaida et al reported the contrary by showing the majority of orphan children having normal nutritional status, with no clear relationship between orphan-hood and the nutritional status of children compared with those who lived with their parents.^13^ Nevertheless, there are some studies done upon children residing in urban slums where prevalence of diarrheal disease and respiratory infections has been reported to be higher than our population.^7,14^ This is attributed to multiple other factors, including poor parental socioeconomic condition and knowledge, and large family size predisposing children to undernutrition and lack of care, besides poor sanitary conditions which are generally absent in orphanages.^15,16^ Access to proper treatment can be improved if the orphanage heads are motivated and empowered, and children are given health education regarding common presenting signs and symptoms, and for prevention of common illnesses.

Many diseases were found to be present due to lack of awareness about disease causation. Further, none of the children knew about the complications of most diseases under study, even when they were suffering from it. We could not find supporting evidence for the level of knowledge and awareness among orphan children regarding general morbidities. Further, the poor nutritional status of the children (discussed elsewhere) may have an impact on their cognition levels which may worsen their awareness and treatment seeking practices.^17^ This presents a unique opportunity to the health professionals for additional research; as well as to design easy to understand messages that would enhance the understanding of disease causation and would go a long way for its prevention. We also found the morbidities to be higher among younger age groups which may be explained by their lower immunity levels and young age as a risk factor for most diseases. Our finding is consistent with reports from studies done on children residing in urban slums in other parts of the country.^12,15^

Treatment seeking practices were found to be unsatisfactory with predominance of self-treatment or using drugs without prescription either from local RMPs or upon pharmacists’ advice. Hospital care was sought for very few cases. This is of concern since most of the morbidities in children are accompanied by frequent complications, thereby increasing the morbidity burden further.^18^ Further, orphanage staff may not be trained to identify development of danger signs which may pose grave risks to the children. While the overall health care utilization for common childhood morbidities like pneumonia has been reported to be only 68% by UNICEF, we could not find any published reports on treatment seeking practices due to which we cannot comment on whether this finding is unique to our setting or generalizable across the country.^3^

## CONCLUSION

Orphanage children represent a marginalized section of the community with special needs. Present study showed a triad of poor knowledge, care and prevention of common childhood morbidities among a sample of South Indian institutionalized orphan children. The burden of common illnesses was high, while awareness and treatment seeking was low.

## Figures and Tables

**Table 1:** Demographic characteristics of orphan children.

Characteristic	Orphaned children n(%)			p
	Boys (n=84)	Girls (n=16)	Total (n=100)	
Mean age±SD (years)	12.59±1.79	11.81±1.79	12.47±1.80	0.16
Proportion school-going, n (%)	63 (75)	9 (56.25)	72 (72)	0.12
Proportion receiving any form of education, n (%)	84 (100)	16 (100)	100	-
Mean duration of living in orphanage±SD (years)	6.2±2.7	5.6±2.2	6.1±2.4	0.40
Proportion engaged in any income generation activity, n (%)	26 (30.95)	7 (43.75)	33 (33)	0.32
Proportion having access to healthcare, n (%)	84 (100)	16 (100)	100	-
Mean height±SD (cm)	142.5±11.20	137.68±7.73	141.74±10.84	0.09
Mean weight±SD (kg)	34.07±9.27	31.06±7.03	33.59±8.99	0.20
Mean BMI±SD (kg/m^2^)	16.62±2.95	16.22±2.72	16.55±2.70	0.60
Undernourished (BMI <18.5 kg/m^2^)	68 (80.95)	11 (68.75)	79 (79)	0.15

**Table 2:** Distribution of common morbidities among orphan children.

Morbidity	Number (%) n = 100	Mean duration of illness±SD (days)	Mean no. of episodes±SD (per year)
Skin infection including fungal infection of nails and scalp	76	12.7±5.2	2.3±0.4
Scabies	46	14.2±8.4	1.8±0.9
Upper respiratory infection	37	2.4±1.1	3.0±1.6
Pneumonia/ lower respiratory infection	4	2.1±0.6	2.3±0.4
Fever	28	1.8±0.9	3.5±1.7
Headache	2	1.2±0.3	2.0±1.4
Pain, other	4	1.9±1.0	2.5±0.8
Diarrhea/ gastroenteritis	26	4.2±2.2	4.7±2.5
Urinary tract infection	1	2.3±0.6	1.4±0.2
Worm infestation with signs of B complex deficiency	21	17.0±8.6	1.3±0.7
Otitis media, acute or chronic	3	5.8±1.2	1.3±0.4
Other ear conditions including wax	74	18.8±11.0	1.1±0.5
Conjunctivitis	2	2.7±0.3	1.0±0.4
Other eye conditions including refractive errors	16	9.3±2.8	1.6±0.7
Injuries	23	1.8±0.3	4.5±1.1
Total*	78	7.0±1.1	1.8±0.2

*Some children were found to have multiple morbidities

**Table 3:** Treatment seeking practices for selected morbidities.

Morbidity	Place of treatment for present morbidity, n(%)			
	Hospital	Private practitioner/ local RMP	Pharmacy, over the counter	Others (including self-care)
Skin infections n = 82	18 (21.95)	27 (32.92)	12 (14.63)	25 (30.48)
Upper respiratory infection n = 37	2 (5.40)	11(29.72)	8(21.62)	16(43.24)
Pneumonia/ lower respiratory infection n = 4	1 (25)	2 (50)	1 (25)	0
Fever n = 28	3 (10.71)	15 (53.57)	0	10 (35.71)
Diarrhoea n = 26	2 (7.69)	5 (19.23)	10 (38.46)	9 (34.61)
Ear diseases n = 77	6 (7.79)	7 (9.09)	13 (16.88)	51 (66.23)
Eye conditions including refractive errors n=18	0	1 (5.55)	4 (22.22)	13 (72.22)
Injuries n = 23	4 (17.39)	9 (39.13)	2 (8.69)	8 (34.78)
